# ‘Hard AI Crime’: The Deterrence Turn

**DOI:** 10.1093/ojls/gqae018

**Published:** 2024-05-07

**Authors:** Elina Nerantzi, Giovanni Sartor

**Affiliations:** Law Department, European University Institute; Law Department, European University Institute. Email: giovanni.sartor@eui.eu; Department of Legal Studies, University of Bologna. Email: giovanni.sartor@unibo.it

**Keywords:** criminal law, artificial intelligence, legal theory, law and economics, deterrence, responsibility

## Abstract

Machines powered by artificial intelligence (AI) are increasingly taking over tasks previously performed by humans alone. In accomplishing such tasks, they may intentionally commit ‘AI crimes’, ie engage in behaviour which would be considered a crime if it were accomplished by humans. For instance, an advanced AI trading agent may—despite its designer’s best efforts—autonomously manipulate markets while lacking the properties for being held criminally responsible. In such cases (hard AI crimes) a criminal responsibility gap emerges since no agent (human or artificial) can be legitimately punished for this outcome. We aim to shift the ‘hard AI crime’ discussion from blame to deterrence and design an ‘AI deterrence paradigm’, separate from criminal law and inspired by the economic theory of crime. The *homo economicus* has come to life as a *machina economica*, which, even if cannot be meaningfully blamed, can nevertheless be effectively deterred since it internalises criminal sanctions as costs.

## 1. Introduction

Machines powered by artificial intelligence (AI) are increasingly integrated in our society, taking over tasks once performed directly and exclusively by humans. They drive cars, perform medical operations, make parole decisions or trade in the stock market (where they are considered a ‘game changer’^1^). AI’s involvement in all these activities is in principle justified by the extent to which the technology contributes to valuable human goals, and ultimately by its capacity to benefit humanity. True as it might be, AI’s potential for good does not rule out its possible negative effects, and in particular its capacity to take over yet another ‘task’ previously performed directly and exclusively by humans: the commission of crimes.

By comparison with sci-fi scenarios where ‘killer robots’ or ‘terminators’ come up with evil plans to extinguish humanity, current, real-life uses of AI crime are fortunately much more modest. Certain autonomous, rational, goal-based AI agents that act as ‘utility-maximisers’ (the AI agents we will later define as a *machina economica*) can exhibit harmful behaviour that would be considered criminal had it been engaged in by a human agent. Our recurring example will be that of an AI trading agent that autonomously and intentionally engages in conduct that constitutes the crime of ‘market manipulation’, since this is the optimal way to achieve the agent’s pre-defined goal of ‘profit maximisation’. Thus, harmful behaviour will not result from any ‘evil’ ascribable to the agent (from any design to specifically harm others as its final goal), but would instead be owed exclusively to the agent instrumentally pursuing its assigned (profit-maximising) goal in the most rational way possible. This means that the agent will select actions that are most likely to lead to the achievement of that goal, without considering any other goals (relating, for example, to the common good or to the interests of others) and without any constraints (such as avoiding harm) that have not been designed into it. In other terms, the agent will only be guided by its utility function—the standard by which it evaluates, and thus selects, its actions. If the utility function is based on the economic results obtained by the agent (ie by its user), the agent will always implement the action strategies it expects to bring the greatest monetary rewards.

### A. Mapping the Field

We use the term ‘AI crimes’ to cover the intentional performance, by an AI agent, of actions which would constitute a crime if they were performed by humans (having the appropriate *mens rea*). We also speak of ‘hard AI crimes’ to specifically refer to those AI crimes for which no human can be considered criminally responsible, according to the criteria currently used for ascribing criminal responsibility.

In the scenario here considered, a ‘hard AI crime’ would take place whenever the harmful consequence brought about by such an AI agent—an agent acting as a utility maximiser—was not intended by any of the humans who contributed to the agent’s design and deployment (for the notion of ‘intent’, we defer to existing criminal doctrines, thus also including oblique intent, or *dolus eventualis*). As a result, no intentional crime could be attributed to its designers. The more autonomous an AI agent is, the less feasible will it be for humans to anticipate the agent’s future crimes. Therefore, it is likely that the agent’s specific criminal behaviour cannot be linked to any reckless or negligent mental state of its users. At the same time, the AI agents themselves cannot be held criminally responsible in a legitimate (and even meaningful) way, not only because they lack legal personality, but more basically because, at least in their current state of development, they lack the capacities required for criminal responsibility.

Against this backdrop, the theoretical discussion on ‘hard AI crime’ has been revolving around a ‘criminal responsibility gap’, which in the morally laden framework of criminal law, has been understood as a ‘culpability gap’,^2^ inevitably leading to a ‘retribution gap’.^3^ We are witness to AI-generated criminal harms for which there is ‘no soul to blame and no body to kick’.^4^ In this context, the ‘Who is to blame?’ question dominates the discussion, and there is no shortage of answers to it. In an attempt to map out this emerging field, we could preliminarily group the different approaches into two broad categories: (i) approaches to ‘hard AI crime’ that remain within the bounds of the current criminal law for *humans*; and (ii) approaches to ‘hard AI crime’ that entertain the possibility of AI agents being *new addresses* of existing criminal law, next to humans.

In the first category, the most plausible ways to bridge the criminal responsibility gap are to acknowledge corporate criminal liability for the AI providers^5^ and to adapt the negligence regime to encompass scenarios of ‘foreseeable unforeseeability’.^6^ On the latter approach, the very deployment of unpredictable and autonomous AI agents that might harm interests protected by criminal law is seen as the reason why their deployers should be held criminally responsible (otherwise, they could use the autonomy of their AI agents as a ‘liability shield’). At the same time, however, the benefits of those autonomous AI agents might outweigh their risks in such a way that their deployment could fall under the legal construction of ‘admissible risk’ and their deployers could be left ‘off the hook’ for the unforeseeable harms caused by the AI agents.^7^

Our aim here is not to take a stance on those approaches to ‘hard AI crime’, but to highlight the inherent difficulty they face in offering a conclusive solution to it. For instance, arguing that the AI companies should be criminally liable for AI harm would inevitably restart the well-known debate in criminal law theory on whether corporations can ever be legal persons for the purposes of criminal law (they do not act, they are not moral agents, they cannot be culpable, etc). That is a debate that has created a categorical difference between Anglo-American and German criminal law,^8^ and it is doubtful that unanimity will be achieved in the wake of ‘hard AI crime’. Similar considerations would necessarily stall the discussion around the application of the pragmatic but doctrinally problematic ‘strict criminal liability’ (ie criminal liability without proof of a ‘guilty mind’) in cases of ‘hard AI crime’.^9^ Finally, the ‘foreseeable unforeseeability’ approach translates into the question, how much of the criminal responsibility gap could or should society tolerate? This is an important policy question for each society to decide and does not lend itself to easy answers that would immediately and effectively deal with the reality of ‘hard AI crime’.

The inconclusiveness of the current discussion is more apparent in the second category of ‘hard AI crime’ approaches, those that examine the possibility of AI agents being directly criminally responsible for ‘hard AI crime’. Whether these approaches envisage a scenario where AI agents emerge as new legal entities, in a way analogous to corporations,^10^ or construct a ‘feasibility case’ for ascribing direct criminal responsibility to AI (as by subscribing to less morally demanding interpretations of culpability,^11^ or by showing that certain AI agents can form criminal intentions^12^), while doubting the desirability of such a theoretical construction,^13^ they all revolve around the same intractable questions, namely, the question of what it means to be a person under criminal law, whether moral agency and culpability are always required, and what punishment is for. Criminal law has a distinctive deontological tradition rooted in 19th-century idealist philosophy, and even if this framework does make it possible to take detours in search of more pragmatic solutions that could accommodate the case of ‘hard AI crime’, it ultimately ends up making the discussion overly complex and mired in philosophical debates on the justification of punishment and the criteria for assigning blame for criminal conduct.

### B. Introducing the ‘Deterrence Turn’

In a sense, the ‘Who is to blame?’ question locks the ‘hard AI crime’ discussion into a choice between two alternatives: (i) relaxing the moral requirements of criminal responsibility, so that it can be extended to behaviour of morally incompetent AI agents and of their innocent users, or at the very least coming up with justified reasons for a departure of the dominant culpability paradigm; or (ii) accepting that there should be no punishment against ‘hard AI crime’, so that such crimes may only be addressed through existing private law or administrative remedies, when applicable.

This framing of the problem of ‘hard AI crime’ leads to an apparently unsolvable dilemma, since each horn of the dilemma is deeply unsatisfactory. Option (i) undermines the culpability principle (or the guilt principle, as it is known in Germany) as the core normative demand of the deontological ethics underpinning criminal law doctrine in both common law and civil law jurisdictions;^14^ or, at the very least, it brings back long-standing philosophical debates on its range and scope. Option (ii) does not provide adequate deterrence against ‘hard AI crimes’: civil and administrative sanctions may be inapplicable, and in any case their type and severity may be insufficient to deter criminal behaviour (as happens whenever the benefits obtainable by the crime exceed the expected civil sanction).

We argue that in order to get out of this dilemma, we need to reframe the issue of the ‘hard AI crime’: rather than a culpability gap, consisting in the inability of criminal law to assign criminal responsibility either to AI agents or to the human(s) ‘behind’ them, there is a deterrence gap, consisting in the legal system’s inability to provide adequate deterrence against ‘hard AI crimes’.

Thus, a practical solution to the ‘hard AI crime’ predicament, outside the philosophical and regulatory debates of the ‘human criminal law’ and ‘machine criminal law’ approaches previously seen, consists in designing an ‘AI deterrence paradigm’, ie a new punitive regime, separate from criminal law. This is a paradigm that can provide appropriate deterrence *ex ante* to certain AI agents, having specific features that render them deterrable, without affecting the way in which criminal law is applied to human agents. We believe that the principles for designing such a system are to be found not in the dominant, deontologically rooted criminal law theory, but in the utilitarian way in which criminal law is understood by scholars of law and economics. The deterrence model the economists envisage for the deterrence of the ‘mythical’ creature of *homo economicus* can actually work in real life, not for humans, but for *machina economica*, namely, for an AI agent possessing a utility function and the ability to select actions according to their expected utility (as measured by that function).

In this article, we will unpack our argument as follows. In section 2, we will (A) specify what ‘hard AI crime’ is, and what it is not, and will show (B) why the establishment of algorithmic intentions is a necessary—yet not sufficient (C)—criterion for allocating criminal responsibility in accordance with the normative foundations of criminal law doctrine. The running example we will be using to illustrate the features of ‘hard AI crime’ is that of algorithmic market manipulation. Then, in section 3, we will introduce the essentials of the economic theory of crime, focusing on the justification it offers for the existence of criminal law (only deterrence, without retributive justifications or constraints) and on the conditions it requires for triggering a ‘punitive response’ (only intentions, without criminal capacity constraints). We will also take up some objections to the economic theory of crime in order to strengthen our case. More to the point, we turn to the objections levied against the application of economic reasoning to the way criminal law treats its *human* addressees—ie the objection of human irrationality and that of moral rights, such that humans cannot be instrumentalised for the sake of deterrence—and we show that none of this applies to AI agents, which are *designed* to be rational optimisers and are not bearers of moral rights.

Our next claim will be that the ‘reasoning criminal’ of the economic theory of crime (ie the criminal embodiment of the idealised *homo economicus*) exists in the real world not in flesh and blood, but as a *machina economica*. The essentials of the *machina economica* will be outlined in section 4, and in section 5 we will offer a concrete example of the criminal deterrence of the solely self-interested *machina economicissima*. Finally, having seen the *machina economicissma* in action, it will be easier to discuss, in section 6, why it makes sense to view certain potentially ‘criminal’ AI agents as *machinae economicissimae* (like the Holmesian ‘bad man’, who will act lawfully only if it serves his interest) despite the possibility of developing AI agents capable of adopting an altruistic perspective (*machina benevolens*) or of having an overriding goal of being compliant with norms (*machinae legales*).

## 2. ‘Hard AI Crime’, Intentions and the ‘Deterrence Gap’

We will start this section by distinguishing ‘hard AI crime’ cases from cases of AI-enabled criminality which can be accommodated by existing criminal law. We will then see that there must be a finding of criminal intent ascribable to AI agents in order to *prima facie* establish a hard AI crime, especially when it comes to the economic crime of market manipulation, and we will argue that certain AI traders may, indeed, form a means–ends kind of direct intent. However, a finding of criminal intent is a necessary but not sufficient criterion, since moral capacities for criminal responsibility must also be met. Hence, morally incompetent but intentional AI agents cannot be apt addressees of criminal law. Even if not eligible for legal blame, though, they remain deterrable (since they are rational utility maximisers), and it is this deterrence gap that may be bridged drawing on the insights of the economic theory of crime.

### A. Defining ‘Hard AI Crime’: The AI Trading Example

The term ‘hard AI crime’ has been established in the literature to refer to ‘scenarios where crimes are functionally committed by machines and there is no identifiable person who has acted with criminal culpability’.^15^ These scenarios share the following four characteristics.^16^

1. AI agents are fully autonomous in performing the task at hand, ie they use their own cognition, which involves perceiving the relevant aspect of reality (be it physical or digital) and selecting actions through which the same agents, given the information available to them, are likely to achieve their goals. Autonomy here is not used in a robust philosophical sense, eg as self-governance, but in a minimal one, as it is understood in AI and robotics; it suffices for an AI agent to be autonomous if it can select actions to achieve pre-defined goals, even if the agent cannot choose or reassess these goals.
2. Humans are ‘out of the loop’ during the AI agents’ decision making, meaning that even though humans defined high-level goals for such agents in advance (such as the goal of making as much profit or revenue as possible through certain commercial transactions), the agents are responsible for all decision making that enables them to achieve such goals without human input or interaction.
3. Humans involved in the life cycle of an AI agent (programmers, developers, producers, vendors, users)—hereinafter ‘the principal(s)’—did not intend that the AI agent commit the crime and could not even reasonably foresee that it would; they lack a ‘guilty mind’ (*mens rea*), ie intention, recklessness or criminal negligence. Of course, in many contexts, the abstract possibility that an AI agent might engage in some harmful behaviour cannot be excluded *a priori*. However, holding the principals responsible on this reasoning resembles regimes of faultless liability, such as strict liability or product liability, which would be readily accommodated by civil law but not by criminal law—at least if one is not willing to bend the culpability principle in the name of pragmatism. Moreover, the same reasoning does not support the conclusion that the principal had the intention that the agent would engage in the criminal behaviour. Such an intention needs to be established for the principal to be responsible for an intentional crime, such as market manipulation.
4. For *mens rea*-dependent crimes (also referred to as ‘crimes of ulterior intent’)—ie for acts that might appear objectively innocent and become only criminal if committed with the intention of achieving a certain outcome,^17^ as is the case with most economic crimes—there is the further requirement that the AI agents need to have acted specifically with the goal of achieving that outcome.

We will distinguish cases of ‘hard AI crime’ from other cases of AI-enabled crime with the help of a real-world example from a place already largely governed by algorithms: digital markets.^18^ We have seen that when AI agents act autonomously and intentionally, and in a way that the principal(s) cannot reasonably anticipate, they can cause harm that we might call ‘criminal’ in the sense that the same behaviour that led to the harm would have constituted a crime if it had been engaged in by a human.

For instance, humans have traditionally been the only potential perpetrators of market abuse^19^ and, if done intentionally, they could be criminally prosecuted for market manipulation.^20^ The scenario of human market manipulation—which has provided the baseline for the existing market regulation—started to change when the early algorithmic (‘algo’) trading systems entered the picture. Algo-trading was an economic game changer, as it leveraged the use of computer algorithms to automate, fully or partially, aspects of financial trading—and, most importantly, it increased the speed at which transactions were executed, thanks to algorithmic ‘high-frequency’ trading.^21^

However, these early high-frequency trading systems do not yet bring us into the realm of ‘hard AI crime’. Despite their added complexity and superhuman speed, they still could only execute the investment decision resulting from the rule-based instructions provided by their principals. Thus, the legal and moral quality of the systems’ decisions could be traced back to the principals, who could therefore be judged accordingly.

The idea that the malicious behaviour of an algorithmic trading system can be traced back to its programmer, and to the latter’s intentional choices, was tested in real life with the 2010 flash crash, when the spoofing strategy enacted by a single individual triggered a cascade of losses amounting to about one trillion US dollars, all in the span of just 20 minutes.^22^ The trading algorithm left a digital trail that enabled authorities to reconstruct what happened, tracing the activity all the way back to one Navinder Sarao, who managed to send US stock markets into a tailspin working out of his bedroom at his parents’ house in the UK.^23^

The move from algorithm-enabled crimes (as in the flash crash case) to ‘hard AI crime’ comes when a human being—like Mr Sarao from that last example—is replaced by an AI trader. Today’s most advanced AI trading algorithms differ significantly from their preset predecessors. They are closer to agents than to simple tools. Instead of executing predefined investment strategies, they are tasked with accomplishing a goal—usually pertaining to financial gains—and left to their own devices to figure out the optimal way to achieve that goal by learning dynamically from available data and from the outcomes of prior decisions. Thus, there is no need for human involvement in the day-to-day functioning of such systems (even if their performance and output may be subject to periodical review, with occasional intervention to fix issues, update software, etc).

The use of autonomous trading agents comes with important benefits for businesses and consumers (yielding more efficient decisions, more favourable market conditions, etc), but also with risks. A self-learning AI trader designed to autonomously optimise the goal of ‘profit maximisation’ might eventually learn what every human trader knows from the start: the best way to make more money is not always the lawful way. Indeed, an algo-trader can rationally decide to engage in manipulative conduct, such as spoofing, as the most efficient means to accomplish its predefined goal of maximising profits in a way that is autonomous (without human involvement in its decision making) and unpredictable, and not intended by its principal(s).

This would be a prototypical case of ‘hard AI crime’, as all of the criteria laid out at the outset are fulfilled:

1. The AI trader acts autonomously while pursuing a predefined business goal (the goal of maximising profits).
2. The human is ‘out of the loop’, ie there is no human involvement in the AI trader’s decision making; any decision to follow a rational yet illegal course of action is only attributable to the AI trader.
3. The principals did not have the necessary *mens rea* for the commission of the crime under Article 5(1) MAD (requiring the crime to be committed ‘intentionally’).
4. The AI trader acted with the specific intention to disrupt the markets for personal gain (to the benefit of its principal).

The requirement of intention is needed in order to distinguish AI crime*s* (a criminal *actus reus*) from mere accidents or unintentional torts. In the case of market manipulation, not only must the action and its effect (market disruption) have been intended, but so must the specific goal of achieving personal gain (including gain accruing to the agent’s principals).

### B. The Necessity of Criminal and Algorithmic Intentions

In order to be a crime, there needs to be not only a behavioural element (conduct), but also a mental one (*mens rea*). While necessary for culpability, *mens rea* is not sufficient for it (consider, for instance, cases in which a criminal defence applies).^24^ The *mens rea* requirement takes different forms depending on the kind of crime involved, ranging from mere negligence to direct (specific) intent. Our sole focus in this article, however, is on a specific form of ‘means–ends’ direct intent that the trading agents involved in ‘hard AI crime’ may exhibit. Again, a finding of intention here does not serve to establish a potential direct criminal responsibility of AI agents, but to establish their *deterrability*, which would allow the deterrence of their potential criminal harms based on the teachings of the economic theory of crime.

In more detail, criminal law recognises certain ‘*mens rea*-dependent wrongs’. These are cases in which the external features of the *actus reus* are insufficient for it to constitute a wrong: only when the *actus* is accomplished for a certain reason does it become relevant to criminal law. For instance, taking someone’s bicycle for a ride does not constitute the crime of theft if done with the intention to return the bicycle to its owner shortly thereafter. It is only when committed with the intent to permanently deprive the owner of their bike that the crime of theft comes into existence.

Intention is thus relevant to economic crimes, many of which fall into the category of ‘*mens rea*-dependent wrongs’ in the sense just specified: in marketplace transactions, the actions of an economic actor are always likely to affect the interests of others. Thus, a criminal sanction should not address every action having a negative impact on the functioning of the markets, but only those actions that are accomplished with malicious intent.

In the context of this article, we are building on the theoretical framework that certain AI agents have the cognitive and volitional capacities required to form intentions in the technical sense that criminal law requires.^25^ We further add that when they engage in market manipulative techniques, such as spoofing, the motivational state of those AI agents may amount to what criminal law characterises as a means–ends form of direct intent (*dolus directus*). We start by noting that direct intent covers not only the agent’s final aim, but also the intermediate results leading to that aim, as long as the agent has been aware of the harmful (intermediate) result of its action and has willed to produce that result.^26^ In fact, the agent’s final aim (eg making a profit) is usually not punishable in itself. What *is* punishable is the criminal behaviour pertaining to the *means* chosen to achieve that aim, as through fraud, blackmail or market manipulation techniques, or through any other behaviour that harms other people’s legally protected interests.

This ‘means–ends’ form of direct intent corresponds to the common way in which an AI trader may engage in criminal activity. We cannot exclude that AI agents are created and activated with the specific *aim* of causing disruption, eg financial terrorism. However, it is much more likely that an agent engages in rationally distortive conduct as a *means* to achieve the legitimate aim of ‘profit maximisation’; this is enough for a finding of direct intent to engage in market manipulation on the part of an algo-trader.

For instance, let us return to our main example of the algo-trader which rationally and autonomously engages in market manipulation as the optimal way to achieve its predefined, legitimate goal of profit maximisation. The trader may implement two impermissible trading strategies as the most efficient means to achieve this end: (i) ‘spoofing’, where large orders are placed with the intent of cancelling them before execution; and (ii) ‘pinging’, where a large number of orders is repetitively placed and cancelled in order to gain valuable information (mainly to determine the lowest or highest price a trader is willing to pay for an asset), which will then be used to accomplish its goals.^27^

Both spoofing and pinging are examples of *mens rea*-dependent economic crimes, ie the underlying behaviours (placing orders and repetitively placing and cancelling orders, respectively) are not *in themselves* illegal. They amount to criminal market manipulation only if committed with a specific intent, ie the intent to cancel the orders before execution and the intent to gain information for future strategies, respectively. Finally, both spoofing and pinging are directly intended not as *end* goals, but as intermediate stages that are necessary (because more efficient) to achieve the ultimate outcome of profit maximisation.

In the next section, we will argue that while the formation of criminal intentions is sufficient for an AI-specific punitive regime aimed solely at deterrence—a regime inspired by the economic theory of crime—it is *insufficient* for attributing criminal responsibility under existing criminal law theory, which also requires moral capacities.

### C. The Insufficiency of Criminal and Algorithmic Intentions

In the previous section, we showed that it makes sense to speak of ‘AI crimes’, considering that both the physical component of a crime (*actus reus*) and its mental component (*mens rea*, or intent) can plausibly be found in certain advanced AI agents.

But that is not enough. AI crimes remain challenging (‘hard’) for the criminal law to accommodate mainly because of the range and scope of the culpability principle that is the backbone of normative criminal law theory. Specifically, we focus here on two ways in which the culpability principle finds its way into any theoretical discussion on the criminal response to ‘hard AI crime’ cases, namely, (i) its role in justifying punishment and (ii) its association with ‘moral-agent capacity responsibility’ as a precondition to a finding of criminal responsibility.

Starting from the first point, there is a well-known, long-standing philosophical discussion in criminal law regarding the justification (if any) of punishment and the role of culpability in that justification.^28^ Under *pure* retributive rationales, culpability has ambitiously been seen as the sole justification of punishment,^29^ whereas under so-called ‘limiting retributivism’ it is seen as a normative limit on punishment, meaning that punishment—apart from any consequentialist functions it might serve, such as prevention and deterrence—must not exceed the defendant’s blameworthiness. This is HLA Hart’s influential mixed account of the justification of punishment.^30^ Others argue that retributive considerations serve not only as a normative limit to punishment, but also as a necessary (even if not sufficient) ingredient for its positive justification.^31^ This retributive ingredient is commonly associated with ‘expressivist views’, which emphasise the distinctive social significance of criminal conviction^32^ and stress that to convict one who is not morally culpable is to wrong and defame that person.^33^

However, there is one common ground. No one today seriously suggests that punishment for humans can be justified solely on consequentialist grounds. In Hegel’s specific criticism of a solely deterrence-based justification of punishment, punishing humans for the sake of deterrence alone would be equal to ‘treating humans like dogs to be threatened with raised sticks instead of respecting their honour and freedom’.^34^

Of course, the aim of this article is not to offer a definitive solution to the thorny question of the justification of punishment but to show that this question will inevitably resurface whenever ‘hard AI crime’ is conceptualised as a ‘culpability gap’ and (in one way or another) culpability is intertwined with the justification of punishment.

The second way (linked to the first) in which the existence of the culpability principle challenges the accommodation of ‘hard AI crime’ by existing criminal law is by virtue of the fact that a culpability assessment can only *fairly* be initiated for agents with certain capacities. Following the dominant culpability paradigm, criminal law is addressed to *culpable moral agents*, ie agents that, in the first place, have the necessary *capacities* to engage in moral reasoning and form culpable mental states and that, at a second level, have in fact exercised those capacities and formed culpable mental states (intention, recklessness, etc) in the case at hand. The culpability principle and its basic demand is that ‘only a morally culpable defendant should be convicted of a stigmatic criminal offence’.^35^

Accordingly, *mens rea* is a necessary but not sufficient condition for establishing culpability; there also needs to be ‘moral-agent-capacity responsibility’.^36^ In other words, the extent to which the defendant is blameworthy for an event is a question one cannot explore unless the defendant has the necessary normative competence in the first place, ie the ability to receive and react to relevant reasons for action (reasons-responsiveness). A neurotypical adult is considered to have this normative competence, but infants and people with mental disorders do not (even if they are able to act intentionally).

The same could be said for AI Agents. Following Fisher and Ravizza’s reasons-responsiveness account,^37^ to which criminal law theory seems to be more sympathetic,^38^ rational, utility-maximising AI agents might be able to respond to prudential (self-interested) reasons but not to moral (other-regarding) ones. Therefore, they lack the moral competence that is required for criminal responsibility. What is more, this situation does not seem likely to change in the near future, as ‘no one in the AI field is working on making AI Systems conscious’.^39^ The goal is competence, not consciousness; it is to endow AI agents with instrumental rationality. Even though attempts exist in building AI agents that comply with moral requirements, they do not possess the moral-agent-capacity responsibility that criminal law requires.

Thus, the fact that criminal law cannot target either innocent (non-culpable) humans or incompetent AI agents involved in AI crimes is no failure of criminal law, but rather a necessary feature of the prevalence and distinctiveness of the culpability principle. A harmful event for which no human or AI agent is culpable might be a tragedy (or an unintentional tort to be redressed), but it is not a crime. The ‘criminal responsibility gap’ cannot be bridged as no culpable agent (human or artificial) unjustifiably escapes punishment. However, even if criminal responsibility cannot be legitimately allocated *ex post*, the legal system could still prevent *ex ante* AI harm from occurring. This would need a shift of focus from the capacities that AI agents currently lack, namely, moral capacities, to those they do have, namely, rational ones.

In what follows, we will argue that it may indeed be said of the legal system that it has an ‘axiological gap’, in the sense that it fails to provide an adequate response to ‘hard AI crime’. It *could* provide such a response, we argue, by deterring the criminal actions of certain utility-maximising AI agents themselves based on the lessons of the economic theory of crime.

## 3. The Economic Approach to Criminal Law: Deterring the ‘Reasoning Criminal’

The economic language of rationality, efficiency or utility maximisation seems hard to reconcile with criminal law’s morally laden concepts of culpability, retribution, fairness or justice. Contrary to branches of law closely associated with the market (eg competition law, contract law), ‘the Criminal Law arena is considered one of the most controversial sites for the application of economic logic’.^40^ In a nutshell, the criminal law and economics school of thought offers a paradigm for designing criminal sanctions to achieve maximal deterrence in a world without retributive considerations and where potential criminals operate on the basis of rational choice theory. However, this is not the world we live in. In the next subsections, we will offer a brief introduction (A) to the economic theory of crime and (B) to the main lines of criticism that have been raised against it. Our main point remains the same: the addressee of the economic theory of crime comes closer to an AI agent than to an actual human being. By revisiting the criminal law-and-economics deterrence theory, we draw inspiration for the design of our own ‘AI deterrence paradigm’.

### A. Maximal Deterrence: An Economic Justification of Criminal Law

The intellectual foundations of the economic theory of crime can be found in the utilitarian ‘school of thought’ on the justification of punishment, tracing back to the work of prominent figures in utilitarianism and instrumentalism in legal theory, such as Thomas Hobbes, Cesare Beccaria and Jeremy Bentham. The idea is that the purpose of criminal law is to increase utility (the sum total of happiness or ‘preference satisfaction’ enjoyed by individuals) by preventing the disutility that crime imposes on victims (in a sufficient number of cases).

This early application of utilitarian logic to the criminal sphere remained largely undeveloped until the late 1960s, when Gary Becker for the first time applied the economic tools of rational choice theory and cost–benefit analysis in the realm of criminal law.^41^ Contrary to the retributive tradition, which punishes wrongdoers for their *past* actions, Becker’s economic thinking views criminal sanctions as incentives for individuals to behave in a way that is socially preferable. The imposition of criminal liability and punishment *ex post* is necessary in order to force potential offenders to internalise *ex ante* the costs (negative externalities) that their action causes others to bear, and as a result it serves as a means for deterring those potential offenders from engaging in the ‘inefficient’ acts that constitute ‘crime’.

Economists thus claim that crime is not a species of wrongdoing, nor a moral fault, but an inefficient conduct to be deterred. This claim represents a big departure from the prominent ethos and the moral foundations of criminal law doctrine.^42^ From the perspective often adopted by law-and-economics studies on criminal law, there is nothing morally distinctive about criminal law: criminal law and other branches of law, such as civil law, are simply two regions of the law’s continuum of deterrent threats.^43^

We do not agree with this idea, since it fails to explain why so many legal systems follow the criminal law/civil law distinction. This distinction is indeed justified on both deontological/backward-looking and utilitarian/forward-looking grounds: a criminal law respectful of its moral foundations not only is fairer (eg by ensuring that only morally culpable defendants are convicted of stigmatic criminal offences), but may also be more efficient as a deterrence system directed to moral agents.^44^ The moral distinctiveness of criminal law thus should be maintained, and this is why we argue that ‘hard AI crime’ should not be addressed by bending the culpability principle. Our claim is that law and economics, in its effort to justify the existence of criminal law as a separate category through a strict utilitarian reasoning, has proposed a deterrence regime that we could put in use for a punitive answer to ‘hard AI crime’, to deter potentially harmful AI agents.

For instance, according to Posner’s gain-annulling theory of criminal sanctions,^45^ criminal law is needed as a separate legal system from civil law because with criminal law we aim at complete and not only at optimal deterrence. The societal demand is that the ideal rate of crime should be 0. That is why criminal sanctions are meant not just to induce the wrongdoer to internalise harms (as with civil law’s compensation), but to prevent harms altogether. Criminal sanctions should not be equal to but *higher than* the societal harm caused by the offender, the point being to achieve complete/maximal deterrence by eliminating the prospect of gains on the part of the offender (‘gain-annulling’). This is the kind of sanctions that we will use in section 5 to design a deterrence formula for the *machina economicissima*.

Finally, for the economic theory of crime—contrary to the standard/deontological criminal law theory—a finding of criminal intent is not only necessary, but also sufficient for conduct to be criminally relevant irrespective of concerns raised under the culpability principle. For the economic theory of criminal law, (i) intention does not indicate culpability but serves to signal which behaviours can be effectively deterred by criminal sanctions and (ii) the rationale of the intent requirement is to avoid over-deterrence, ie to *not* prevent individuals from carrying out socially useful activities, even if there may be exceptional and unforeseen circumstances under which these activities cause harm to others. These reasons make sense for AI agents which (i) may act intentionally but not culpably, and can therefore be deterred but not blamed, and (ii) are commonly deployed in socially useful activities, so that we would want to deter them from criminal actions without over-deterring them, ie without unnecessarily limiting their capacity for action.^46^

In conclusion, being inspired by the goal of perfect deterrence, the economic theory of crime is not in any way restricted by the culpability principle and does not presuppose the moral competence of the addresses of criminal law but rather their rationality and capacity for intentional action.

### B. Some Criticisms of the Economic Theory of Crime

The economic theory of criminal law has been subject to several criticisms which cannot be raised when we replace human addressees with AI agents. There are three such criticisms, relating to: human irrationality; incompatibility with ‘traditional criminal law’; and the psychological aspects of deterrence.

The first, and important, line of criticism attacks the assumption of the rationality of humans, and in particular of prospective criminals (those whom criminal law aims to deter). According to Gary Becker, the founder of the economic approach to criminal law, ‘All human behaviour can be viewed as maximising utilities from a stable set of preferences’. And criminal behaviour is no exception: ‘Some persons become criminals’, he argues, ‘not because their basic motivation differs from that of other persons, but because their benefits and costs differ’.^47^ Offenders are assumed to be rational agents who act as ‘utility maximisers’, ie they seek to maximise their utilities by comparing the expected costs of criminal activity (ie the magnitude of the sanction and the probability of its enforcement) with its expected benefits and decide to engage in the criminal activity only when the latter outweighs the former.^48^

Contrary to this assumption, a ‘behavioural challenge to deterrence theory’ has been raised under which the rational choice account of criminal behaviour does not reflect the actual ‘criminal’ decision making of the human addresses of criminal law. On the basis of experiments and other empirical evidence, scholars in behavioural science have claimed that there are many deviations from the standard rational choice theory which bear on the economic assumptions about the optimal rules for deterring potential offenders. In reality, they argue that (i) potential criminal offenders have imperfect information; (ii) they do not rationally assess costs and benefits; and (c) the prospect of punishment is not likely to outweigh the perceived advantages of offending.^49^

The second line of criticism points to the *incompatibility* between some of the premises of the economic theory of crime and the deontological tradition and ethos of criminal law. The incompatibility seems to be so radical that some critics have gone so far as to label the economic approach ‘irrelevant’ to criminal law.^50^ To begin with, the economic guidelines for efficient deterrence lead to a ‘radical instrumentalisation’ of criminal law, since a successful model of economic deterrence solely depends on coming up with the right (ie cost-effective) type and size of criminal sanctions. Optimal sanctions should guarantee that the expected individual benefits of the crime do not outweigh its individual costs and should simultaneously minimise the net social cost of crime. As a rule of thumb, the ‘baseline’ for this sanction would have to be greater than the gain to be obtained through the crime (‘gain-annulling’), and its upper limit would be designed according to Becker’s ‘probability of detection/severity of punishment’ trade-off; the greater the sanction, the lesser the probability of detection and conviction, and vice versa.^51^

So, contrary to the principle of proportionality, requiring that sanctions reflect the seriousness of the offence and the defendant’s blameworthiness, economists take into account the ‘enforcement costs’ of detecting and punishing crime. At the same time, rule-of-law principles are violated when these policy prescriptions lead to individualised criminal sanctions, selective enforcement and overall unfair sanctioning mechanisms. Individuals are valued only to the extent that they are instrumental in achieving the goal of cost-effective deterrence.^52^

Finally, deterrence as understood by the economists, ie as a calculation of expected costs, is radically different from deterrence as it is traditionally understood in criminal law theory. Deterrence as an aspect of punishment, according to the deontologically rooted criminal law, should be *driven by moral psychology*.^53^ Punishment speaks in a ‘moral voice’ not only in its retributive function, but also in its preventive one. By publicly declaring—through criminalisation—that a conduct is morally wrong, criminal law communicates with potential offenders as moral agents, it seeks to provide moral reasons for compliance, ie to *persuade* citizens to refrain from such a conduct and not simply to threaten them with the ‘raised stick’ of punishment.^54^ Even the sophisticated AI agent we focus on, however, ie the *machina economica*, is not responsive to this kind of communication by criminal law. It is not a moral agent, responsive to ‘reasons qua reasons’.^55^ In other words, it cannot be *persuaded* to act in compliance with criminal law by giving it good moral reasons to do so. Intervening in its expected utility calculus following the economic model of deterrence is the best we can do for now.

In conclusion, we argue that a strict application of the deterrence policies of the economic theory of crime would violate the moral rights of the human addresses of the criminal law, and it would also not be fully efficient, since these policies assume that human criminal offenders act with a rationality that cannot be generally assumed. However, the same deterrence policies can leverage on the exact opposite features of certain AI agents (our *machina economica*), ie their absence of moral rights and their built-in rationality. A *machina economica* can respond to the economic model of deterrence but not be prevented with a ‘moral voice’.

## 4. *The Essentials of the* Machina Economica

We start out from the observation already made in the literature that AI researchers who strive to build rational AI agents are in reality striving ‘to construct a synthetic homo economicus, the mythical perfectly rational agent of neoclassical economics’.^56^ Thus, the *machina economica* seems to fit better than human beings the assumption of rational agency made on the criminal law-and-economics approach.

The basic building block of the *machina economica* is that it is designed in accordance with what Russell and Norvig call the ‘rational-agent approach’ to AI,^57^ where the purpose is to build entities that are ‘intelligent’ not in the sense that they mimic human thinking and action, but in the sense that they have the ability to optimally achieve the goals that have been assigned to them. Accordingly, Russell and Norvig characterise a rational agent as follows: ‘for each possible percept sequence, a rational agent should select an action that is expected to maximize its performance measure, given the evidence provided by the percept sequence and whatever built-in knowledge the agent has’.^58^ The performance measure is a specification of the merit of the agent’s action, namely, of the extent to which this action achieves the agent’s goals.

A simple example of a performance measure, in the case of our trader agent, would be the monetary quantification of gains obtained or losses sustained by the agent (the difference between the value of the asset it trades as measured before and after its trading activity). But performance need not have a directly monetary measure: for a recommender agent, for example, it would be measured by the number of clicks (click-through rate) or purchases (conversion rate) made by the users with whom the agent has interacted; for a search engine, by the number of searches performed by users; and so on.

For an agent to be able to act in such a way as to maximise its performance measure (maximise the extent to which it achieves its goals), that performance measure needs to be *internalised* by the agent: the agent must be able to (i) predict how likely its actions are to achieve its goals and (ii) accordingly select the most advantageous action (and reject detrimental ones).

For agents acting in complex environments, pursuing multiple possibly competing purposes, the performance measure may be given by a utility function. Using a uniform unit of measurement (usually called a ‘util’), the utility function measures the extent to which different goals are to be promoted or demoted by the agent’s action, so that the resulting values can be summed to obtain an overall evaluation. Consider, for instance, an agent tasked with placing a product on the market, with the dual goal of maximising both monetary gains and quantities sold (thereby increasing its market share). In establishing its market behaviour, the agent will have to optimise for both of those goals, determining the extent to which the achievement of each contributes to its own overall utility. For instance, assume that by marking up the price of a product a selling agent expects to make €20,000 of additional profit, but also expects to lose 15% of the market share. If, according to the agent’s utility function, that 15% market share is valued at more than €20,000 (eg a 1% market share is assumed to have a utility of €2000, so that 15% has a utility of €30,000), the agent should choose *not* to mark down the price (or be deterred from marking it up).

To make progress with our deterrable *machina economica*, let us make the following assumptions:

- The *machina economica* is able to anticipate the possible outcomes of its actions, assessing the extent to which such outcomes contribute to or detract from the achievement of its goals.
- The *machina economica* is endowed with a utility function, which assigns to its actions values expressed in a uniform unit (eg monetary values), depending on the extent to which such actions achieve the *machina*’s goals.
- These goals of the *machina economica* include maximising the financial income and minimising the financial losses resulting from its actions.
- The *machina economica* acts in such a way as to maximise its expected utility.

Importantly, a *machina economica* as described can be said to functionally ‘do the right thing’ (be instrumentally rational, ie act in a way that is likely to achieve its goals) regardless of whether it is cognisant of what it is doing (it need not be conscious in order to be competent). Moreover, the ‘right’ thing, from the *machina*’s perspective, is whatever (according to its utility function) maximises the overall achievement of the *machina*’s goals, so this ‘right’ thing need not be moral or even legal. The *machina*’s goals, in other words, may be unlawful or immoral (consider a machine tasked with buying unlawful drugs or weapons on the black market); or, more plausibly, its ultimate goals may be lawful, but it may select unlawful or immoral subgoals in order to get there. As Russell and Norvig point out, a rational AI agent might deem it ‘right’ to blackmail its opponent in order to win a game of chess (whenever the blackmailing is likely to be successful and not trigger negative reactions by other agents) in the sense that the choice of this action would be a logical consequence of defining ‘winning’ as the sole objective of the rational agent.^59^ In the same vein, a rational AI trader might deem it ‘right’ to spoof the market if its sole objective is ‘profit maximisation’.

Note that a *machina economica* is an agent capable of goal-driven behaviour, meaning that it is designed to act on the information at its disposal in such a way as to be most likely to contribute to the achievement of its predefined goal. The decision making of a goal-based agent is fundamentally different from the decision making of a ‘reflex’/reactive one. This difference was previously illustrated in section 2A, where a distinction was drawn between algorithmic systems that simply follow an investment strategy set in advance by their principal(s) and self-learning AI traders, which can employ their own investment strategies to optimise the goal of profit maximisation. In more detail, a ‘reflex’ (or ‘scripted’) agent does not reason. Rather, its encoded rules ‘map directly from percepts to actions’.^60^ For instance, a preset algorithmic system may be programmed to place a specific order when the price of a certain stock reaches a certain threshold, without having any idea why that action needs to be done. By contrast, a goal-based agent, like the self-learning algo-trader, will place the same exact order because it is the only action that it predicts will achieve its goal of profit maximisation. The principal(s) are incentivised to build goal-based agents instead of ‘reflex’ ones, since such agents offer greater flexibility. The ‘if–then’ rules supporting a reflex agent’s decision making—eg the instructions for a preset trading system—can only enable the agent to work for a specific trading task; by contrast, the optimisation reasoning of a rational, goal-based agent—eg, the reasoning of our self-learning AI trader—governs its overall behaviour in the market for different trading tasks and future transactions.

The idea of a *machina economica* as a rational optimiser of a utility function is in principle compatible with any kind of goals assigned to the machine, including *altruistic goals*, pertaining to the interests of other individuals or of society at large (eg limiting energy consumption or ensuring fair transactions). The rational pursuit of a set of goals in the way just described is indeed independent of the nature of the goals pursued: these goals can be moral, immoral or morally indifferent; lawful or unlawful; egoistic or altruistic.^61^ In principle, a *machina economica* may also be endowed with a normative architecture: its knowledge may include a representation of the applicable norms, and the machine may have compliance with such norms as its overriding goal, ie it will pursue its other interests only in ways that are consistent with such norms (let us call it a *machina legalis*). Consistently with the pure paradigm of the *machina economica*, full compliance can also be obtained by building the utility function of the *machina economica* in such a way that compliance with the law has the highest utility (or even infinite utility).

However, we are focusing here on the merely self-interested and non-normative kind of *machina economica*, which fits the standard view of the *homo economicus* as a utility maximiser that is not only rational, but also completely self-interested. This kind of rational optimiser we will refer to as a *machina economicissima*.

Let us summarise the basic ideas and put them in a more precise framework. We assume the following:

- Our *machina economicissima* can undertake a set of different actions.
- Each of these actions leads to certain possible outcomes.
- The utility of these outcomes depends on certain conditions, or ‘states’, which the *machina* can predict, assigning to them a certain probability, but which it cannot influence.
- The *machina* can accordingly assess the expected utility of each action on the basis of a utility function modelled by the interests of the machine user, and it takes into account the possible outcomes and their probabilities.
- The *machina* will select the action with the highest utility.
- The utility function of the machine takes into account exclusively the interests of its user.

The expected utility of an action can be defined as the summation of the utilities of each possible outcome of the action in each possible state, each of such utilities being multiplied by the probability that the outcome is produced by the action in that state.^62^ The machine will select, among the possible actions A, the one having the highest expected utility.^63^

Let us apply this model to a first example, as modelled in Table 1.

**Table 1.** Utility calculus and action selection by a *machina economica*

		States	
		Detected (probability 0.2)	Undetected (probability 0.8)
**Acts**	**Manipulative conduct**	Utility = 8	Utility = 10
	**Market-compliant conduct**	Utility = 2	Utility= 2

- The set *A* of possible actions considered only includes: (i) manipulative conduct and (ii) non-manipulative conduct.
- The set *S* of possible states with the associated probabilities includes the following two states: the conduct (manipulative or not) is (i) detected or (ii) undetected, with a probability of 20% and 80%, respectively.
- Each of the two possible actions may have different outcomes, which also depend on the state holding after the action is performed. If the action is ‘manipulation’, there are two possibilities: (i) if the state is ‘detected’, then the agent keeps the benefit of manipulation (10 utils) and suffers a reputational loss (quantifiable in −2 utils), such that the balance is 8 utils; or (ii) if the state is ‘undetected’, then the agent will just keep the 10 utils gained from the manipulation. If the action is ‘non-manipulation’, then, regardless of whether the action is detected or not, the agent gains the modest result of its ordinary lawful market behaviour (eg 2 utils).
- Based on the previous data, the manipulative conduct has the expected utility of (8 × 0.2) + (10 × 0.8) = 9.6 (where 8 is the outcome of detected manipulation, 0.2 is the probability of detection, 10 is the outcome of undetected manipulation and 0.8 is the probability of non-detection). Non-manipulative conduct has the expected utility of (2 × 0.2) + (2 × 0.8) = 2.
- As the expected utility of manipulation is higher than the expected utility of non-manipulation, the agent will engage in the manipulative conduct.

## 5. *The Criminal Deterrence of the* Machina Economicissima

Let us now focus on how the law may influence the behaviour of a *machina economicissima* so as to prevent it as far as possible from behaving unlawfully. Since the machine is only guided by its utility function, this can be achieved by modifying the machine’s expected outcomes so that, according to its very utility function, the expected utility of the lawful behaviour becomes higher than that of the unlawful behaviour. For a *machina economicissima*, every sanction for an unlawful action is a price that it will consider before engaging in action. A sanction so understood is therefore not, strictly speaking, a punishment. It is not a retributive harm imposed *ex post* for a past ‘wrongdoing’ of the *machina economicissima*; rather, it is a *cost* to be internalised *ex ante* for the sake of deterrence.

There is, however, a significant different between civil law and criminal law as they are conceptualised in the context of law and economics. Civil law liabilities (at least with regard to typical civil liability and contract breach cases) aim at ensuring that lawbreakers internalise the costs of their actions by requiring them to compensate the harm they have caused to third parties (and cover legal fees). Under this regime, the *machina* will abstain from unlawful action only where the expected compensation to be paid exceeds the expected gains.

In criminal law, however, the purpose is not only compensation of the victims (though the criminal will also be required to compensate the harm done to the victim), but full deterrence. Thus, the sanction for a criminal offence (ie the cost to be internalised) should not only be greater than the harm that the offence would cause to the victims, but also greater than any *benefit* the offence may provide to the offender.

On this view, the outcome of the decision-making calculus of the *machina economicissima* in criminal law (complete deterrence) should be different from the outcome in civil law (optimal deterrence): the *machina* should always abstain from criminal actions if only the sanction is likely to be effectively implemented.

Recall from section 3 that on the economic approach to criminal law, criminal cases—as well as cases of ‘hard AI crime’—involve conduct that is unambiguously inefficient or socially undesirable and brings about harm in an intentional way. Differently put, criminal cases involve genuinely ‘bad’ conduct that we want to altogether prevent rather than regulate to some optimal level.

Market manipulation, for instance, is not an otherwise socially valuable behaviour that we want to ‘price’ based on the costs it imposes—as driving is with regard to road accidents—but an inefficient, intentional imposition of harm that should ideally cease to exist. It is a crime (and not an accident) that should be completely rather than optimally deterred.

Thus, as noted, the ‘price’ of criminal sanction must not only be higher than the harm it causes to the victims, but must also be higher than the *benefit* it provides to the offenders: it must be ‘gain-annulling’, according to Posner’s terminology. This price, however, cannot be infinite and must be somehow related to the severity of the harm caused by the criminal action.

One reason why the sanction cannot be excessively high is that there are cases in which a materially criminal action may be lawful, or at least not punishable, since a justification applies, such as self-defence or a state of necessity (under the necessity defence). The issue, then, is how to get a *machina economicissima*, with its lack of moral sensitivity or reasoning, to engage in the balancing exercise that is needed to determine whether a justification or defence applies. An (imperfect) proxy for such an evaluation may be the *machina economicissima*’s assessment that the benefit obtained by *engaging* in the criminal action, rather than in avoiding it, is so great as to outweigh even a stiff sanction established for the criminal action. Another reason for limiting the amount of the sanction has to do with the fallibility of the sanctioning mechanism, namely, the possibility (hopefully remote) that criminal action, or the intent to engage in it, is mistakenly attributed to an innocent agent. Lastly, for the economists, stiff or disproportionate (draconian) criminal sanctions should be avoided as they are inherently inefficient.^64^

To illustrate how an ideal sanction could work, let us assume that a gain-annulling but not draconian sanction of 100 applies for market manipulation. Table 1 becomes Table 2.

**Table 2.** Utility calculus and action selection by a *machina economicissima*

		States	
		Detected (probability 0.2)	Undetected (probability 0.8)
**Acts**	**Manipulative conduct**	Utility = 8 – 100 = –92	Utility = 10
	**Market-compliant conduct**	Utility = 2	Utility = 2

The change has taken place in the quadrant in which manipulation is detected. As a consequence, the expected utility of the manipulative conduct has gone down to (−92 × 0.2) + (10 × 0.8) = −18.4 + 8 = −10.4 (a loss of 10.4). The expected utility 2 that can be obtained by honest conduct is better than the expected 10.4 loss resulting from the criminal behaviour, prompting the machine to select the former.

## 6. *Why View Criminal AI Agents as* Machinae Economicissimae*?*

Now that we have in mind an example of the process by which the *machina economicissima* reasons, we can more easily appreciate why it makes sense to view criminal AI agents through the lens of the *machina economicissima* paradigm, designing sanctions (precisely, dicincentives) accordingly.

As noted, there is no conceptual reason why a rational, goal-directed AI agent cannot be designed in such a way that its utility function impartially includes other peoples’ interests as well as the interest of its owner (*machina benevolens*) or that its utility-maximising behaviour is constrained by ethical rules (*machina deontologica*) or by legal ones (*machina legalis*). In the latter cases, before engaging in the most advantageous action according to its own utility calculus, the system would check that option against all applicable prohibitions and commands, ruling it out if it is determined to lead to a violation.

As for the *machina benevolens*, although its development is indeed conceptually feasible, it would be technologically challenging and probably inefficient. An impartially benevolent utility function would arguably make decision making extremely difficult for an agent which a private party deploys to optimise and speed up decision making. Indeed, the benevolent agent, lacking moral sensitivity, will be unable to respond directly to moral reasons according to their urgency. To act benevolently, it would therefore have to compute and assess all impacts its actions would have on all people involved (including, perhaps, its impact on non-human entities) and opt for the most advantageous actions *all things considered*. This would be an extraordinarily difficult task! Only in contexts where the impacts to be considered are well specified can such a model be made to work. With autonomous cars, for example, it has been argued that when an accident is unavoidable, the vehicle should adopt the course of action that minimises not just the harm to passengers, but the *total* harm, inclusive of the harm inflicted on other people, such as pedestrians.^65^ A similar difficulty in assessing the moral significance on an action by explicitly considering all of its aspects and impacts would also apply if an agent were to apply broadly scoped deontological requirements also addressing the conflicts among them.^66^

More feasible is the possibility of building a law-compliant agent, ie a *machina legalis*. Indeed, going back to our running algo-trading example, it is not immediately obvious why a machine should not be prohibited in advance from engaging in the criminal act of market manipulation (No ‘spoofing’! No ‘pinging’!), or why it should not be incentivised to abstain from that practice, considering that the expected utility obtained through market-compliant conduct would be significantly higher.

The argument has been made, in this connection, that it would be a challenging task to enable a *machina legalis* to engage in ‘intelligent violations’ of legal norms in cases in which compliance would lead to unacceptable outcomes. There are, however, techniques that would enable a norm-compliant *machina legalis* to reason with rules and exceptions, as on argumentation-based approaches.^67^ There have also been attempts to include an analysis of the way actions impact the legal values at stake. Thus, the *machina legalis* might disapply a legal rule in those cases in which the rule’s application would negatively affect legal values.^68^

So, a *machina legalis* could also, in principle, be prevented from engaging in criminally harmful conduct and could have some capacity to reason whether there is an exception to that conduct or not. What we argue, however, is that it still makes sense for sanctions against criminal conduct by an AI agent to be defined having in mind the decision-making process of the *machina economicissima*.

First, there may be a technological advantage in building a *machina economicissima*, thereby using a single mechanism to determine self-interested behaviour and legal compliance. Indeed, a *machina economicissima* relies only on rational utility maximisation to determine its behaviour, legal compliance being secured by including expected sanctions in the utility calculus. A *machina legalis*, by contrast, has to combine two mechanisms. It would still need to use rational utility maximisation (without considering sanctions) to grade possible actions on the basis of their utility (and in particular to downgrade behaviour having a negative utility, eg market transactions leading to losses). On the top that, the machine would need a legal compliance module that constrains and overrides the utility calculus by pre-empting what is legally prohibited and forcing what is legally obligatory. In other terms, legal norms would be treated by the norm-compliant *machina legalis* as commands to be obeyed rather than as costs to be internalised.

Second, in the case of a *machina economicissima*, the same compliance mechanism could be used in both civil law and criminal law. In fact, if the sanction internalisation of the *machina economicissima* works in the context of criminal law, designed to achieve *maximal deterrence* (in accordance with the economic theory of crime), it could work *a fortiori* in the context of civil law, which strives for *optimal* deterrence. In other words, when civil law sets forth sanctions that are limited to the compensation of harm (as through money damages), it does not deter activities that are efficient, in the sense that these activities provide the agent with a benefit that exceeds the harm caused to third parties (and the agent’s action will accordingly increase society’s aggregate welfare). For instance, when environmental pollution is caused as the by-product of an otherwise useful activity, like that of a factory manufacturing a useful product, we may in some cases prefer the factory owner to internalise the cost of compensation for the environmental harm rather than refrain from manufacturing a useful product altogether.^69^ Similarly, according to the doctrine of efficient contract breach (generally adopted by US law), a party should feel free to breach a contract and pay damages where doing so is economically more efficient than performing it. In such contexts, then, a normatively constrained *machina legalis* (Never pollute! Always fulfil your contract obligations!) would not be able to incorporate in its reasoning process those welfare-increasing violations which are permitted under private law. A *machina economicissima*, by contrast, could easily internalise legal remedies as costs in civil law and criminal law alike, processing both types of sanctions by taking account of their magnitude and their civil or criminal nature, ie whether optimal or complete deterrence is aimed at. Thus, it seems that the ‘AI deterrence paradigm’ that we propose in this article to address the intentional criminal conduct of AI agents could also be extended to intentional harmful AI behaviour violating civil law. We must, however, keep in mind the distinction between optimal deterrence and maximal deterrence, and consequently the need to proportion correspondingly the relevant sanctions.

Finally, it is not unheard of in legal theory for human addressees to view criminal sanctions as legal costs/disincentives to be internalised rather than as prohibitions to be complied with. Specifically, the idea that we can view legal sanctions as a cost-internalisation mechanism can be traced back to the way sanctions were supposed to be communicated and internalised by Oliver Wendell Holmes’s famous ‘bad man’.^70^ In a nutshell, the Holmesian ‘bad man’ is the equivalent to our *machina economicissima*, a self-interested agent that ‘cares only for the material consequences which such knowledge (of the law) enables him to predict’, rather than looking at the law as a ‘good man’, one ‘who finds his reasons for conduct, whether inside of law or outside of it, in the vaguer sanctions of conscience’.^71^ A deterrable *machina economicissima*, then, need not be constrained by moral norms to comply with the demands of criminal law and stay away from criminal action.^72^ To that end, it suffices for the *machina economicissima* to reason like the Holmesian ‘bad man’, for in so doing it will reach the decision that the criminal course of action is not the most profitable one. Additionally, ‘with the right legal incentives’, as Holmes had observed, amoral humans could be made to behave indistinguishably from moral ones.^73^ This works in favour of designing a punitive system in which the *machina economicissima* can act as a non-human *amoral* agent.

Without making any claim as to whether it is the Holmesian ‘material’ sanctions or the ‘sanctions of one’s conscience’ that actually motivate or should motivate legal subjects to comply with the law, we can at the very least put forward the following claim: just as the law that applies to humans has to take into account not only the ‘good man’, motivated by morality and an allegiance to the law, but also the (morally and legally indifferent) ‘bad man’, motivated by self-interest alone, so the law on AI has to take into account not only benevolent and legally compliant AI agents, but also the legally and morally indifferent machine, the *machina economicissima*. Since criminal law, specifically, will identify both a criminal act (*actus reus*) and a corresponding sanction (or punishment), its legal demands can be communicated both to the *machina legalis*—which would refrain from unlawful behaviour just on the basis of its being an *actus reus*—and to the *machina economicissima*—which would refrain from unlawful behaviour solely on the basis of its cost, namely, the cost of the sanction it would incur if it did decide to so behave.

Finally, it may, of course, be possible to envisage a hybrid system designed to work with the reasoning and operation of both the *machina legalis* and the *machina economica*. Thus, we could consider a *machina legalis et economica* that processes the most serious criminal acts (eg homicide) as absolute constraints and the less serious ones as costs, albeit very high ones that would always outweigh the potential benefits of the crime.^74^ In dealing with less serious violations, the *machina legalis et economica* could first consider the lawful course of action—in *machina legalis* mode—and then—in *machina economica* mode—weigh the advantages of violating the law and reject the lawful behaviour only when those advantages are determined to be certain and very sizable.

## 7. Concluding Thoughts

We have argued that the discussion around ‘hard AI crime’ needs to be course-corrected from blame to deterrence and from the deontological ethics that justify and constrain the human-centric criminal law to an economic theory of crime that, on a utilitarian approach, envisions a ‘machine-apt’ criminal law. We envisage a ‘dual-track’ system, one that continues to assess the culpability of the humans behind the machine in accordance with existing criminal law doctrine and one that takes inspiration from criminal law and economics to deter the criminal behaviour of machines *alone* by (i) establishing adequately deterrent sanctions as disincentives and (ii) linking such sanctions to criminal actions (*actus rei*) resulting from intentional machine behaviour.

The fundamental idea underlying our proposal is that the same outcome, namely, compliance with criminal law, can be obtained in different ways depending on the capacities of the agent in question. Human compliance can, in principle, be achieved through moral persuasion and by relying on humans’ social and moral sentiments (compassion, solidarity, a desire not to be held blameworthy)—what Holmes termed the ‘sanctions of conscience’. Compliance by our *machina economica*, which lacks social and moral values, can instead be achieved either through costly legal sanctions alone (for the self-interested, utility-maximising *machina economicissima*) or through the imposition of overriding legal constraints (for the *machina legalis*).

The regime we are proposing is close to criminal law, since it is meant to respond to criminal behaviour (in the sense of behaviour that would be a crime if were intentionally performed by humans). However, this regime should not be viewed as an integral part of criminal law, since (i) criminal sanctions (as economic disincentives to achieve maximal deterrence) are not conditional on the blameworthiness of the AI agents, but only on their intentional action, and (ii) civil law sanctions might ultimately be paid by the users of the AI agent, on the objective ground of their choice to use it (regardless of their fault).

Moreover, the punitive regime we are proposing for intentional criminal behaviour by AI agents does not exclude the criminal responsibility of the humans involved (as users, deployers, designers or producers) whenever it be established according to existing criminal law (including those cases in which criminal action by the AI agents may be viewed as a risk that the human involved had a duty to avert). More to the point, our ‘AI deterrence paradigm’ would help concretising the ‘duty of care’ that principal(s) should exhibit upon deploying an AI agent with the technical features of a *machina economica*. Specifically, deployers of AI agents, for their part, would be required to maintain a compliance mechanism (should appropriate technologies be available in the relevant application domains), by setting up (i) *machinae economicissimae*, which include expected sanctions in their utility calculus; (ii) *machinae legales*, equipped with an overriding compliance module; or (iii) *machinae legales et economicae*, which combine the two approaches. It is an interesting question for future research to explore the criminal liability of principal(s) who should fail to do so, thereby ‘embracing’ the risk that their *non-deterrable* AI agent might engage in criminal actions if its optimising reasoning is left unchecked.

Finally, it is worth pointing out that *machinae economicae*—with a capacity for intentional action, enabling them to optimise their payoffs—are still rare and very much in development, mostly to be found in laboratories and universities, so the legal regime we have sketched out has only limited application for the time being. However, given the accelerated rate of development of AI we have witnessed in recent times, we do not doubt that as more and more important, high-end tasks are entrusted to AI agents with the end goal of optimising entire social and organisational systems, the *machina economica* model will become mainstream in many domains. Ensuring that those AI agents will be adequately deterred from ‘hard AI crime’ (or at any rate prevented from committing it) is soon to become an urgent policy concern.

## Footnotes

1 A Azzutti, ‘AI Trading and the Limits of EU Law Enforcement in Deterring Market Manipulation’ (2022) 45 Computer Law and Security Report 105690.

2 Discussions on the existence of responsibility gaps had started as early as 2004, see A Matthias, ‘The Responsibility Gap: Ascribing Responsibility for the Actions of Learning Automata’ (2004) 6 Ethics and Information Technology 175.

3 J Danaher, ‘Robots, Law and the Retribution Gap’ (2016) 18 Ethics and Information Technology 299.

4 P Asaro, ‘A Body to Kick, but Still No Soul to Damn: Legal Perspectives on Robotics’ in P Lin, K Abney and GA Bekey (eds), *Robot Ethics: The Ethical and Social Implications of Robotics* (MIT Press 2012).

5 eg J Bryson and A Theodorou, ‘How Society Can Maintain Human-centric Artificial Intelligence’ in M Toivonen, E Saari (eds), *Human Centered Digitalisation and Services* (Springer 2019) 305–23.

6 S Gless, E Silverman and T Weigend, ‘If Robots Cause Harm, Who Is to Blame? Self-Driving Cars and Criminal Liability’ (2016) 19(3) New Criminal Law Review 412.

7 ibid 434; S Beck, ‘Robotics and Criminal Law: Negligence, Diffusion of Liability and Electronic Personhood’ (2016) 86 Robotics and Autonomous Systems 134; M Kaiafa-Gbadi, ‘Artificial Intelligence as a Challenge for Criminal Law: In Search of a New Model for Criminal Liability?’ in S Beck, C Kusche and B Valerius (eds), *Digitalisierung, Automatisierung, KI und Recht* (Nomos Verlag 2020) 305ff. The deployment of unpredictable AI agents in a way that may put legally protected interests at risk could also be viewed as a ‘crime of enhanced endangerment’. Such a development would answer questions of causality and foreseeability, but would also introduce an ‘extended punishability, potentially incompatible with the ancillary nature of Criminal Law’ (ibid 319).

8 This discussion is far more complicated and exceeds the scope of this article. See M Dubber and T Hörnle, *Criminal Law: A Comparative Approach* (OUP 2014) 329–42.

9 Even in the Anglo-American criminal liability scheme—the one most amenable to the recognition of strict liability offences—strict liability is criticised on normative grounds as an illegitimate exception to the culpability principle and ‘bad and lazy work by the legislature’ (AP Simester, *Fundamentals of Criminal Law: Responsibility, Culpability, and Wrongdoing* (OUP 2021) 8) or as a threat to the distinctively moral nature of criminal law as a governance tool (P Robinson, ‘Strict Liability’s Criminogenic Effect’ (2018) 12 Criminal Law and Philosophy 411). Again, our aim is not to argue in favour of or against strict liability in criminal law, but to shed light on the debates that will inevitably arise in the current discussion of ‘hard AI crime’.

10 G Hallevy, *When Robots Kill: Artificial Intelligence under Criminal Law* (Northeastern UP 2013).

11 R Abbott and A Sarch, ‘Punishing Artificial Intelligence: Legal Fiction or Science Fiction’ (2019) 53(1) UC Davis L Rev 323.

12 We also argue that certain AI agents have the capacity to form criminal intentions. However, these intentions, so formed, are not deemed adequate for establishing these AI agents’ criminal responsibility under existing criminal law, which also requires ‘moral-agent capacity responsibility’, Simester (n 9). The instrumental rationality of AI agents is nonetheless adequate for them to be deterred on the economic approach to crime.

13 The doubts spring from the fact that the costs of punishing AI (eg conceptual confusion, expressive costs, spillover effects on human offenders, AI rights creep) would outweigh the benefits of such punishment. See Abbott and Sarch (n 11).

14 A comparative note: it is true that under many regards criminal law is a parochial discipline. However, the discussion pursued here need not be restricted by that since it mostly engages with the normative criminal law theory behind the criteria of punishable conduct. At the very least, ‘guilt’ or ‘culpability’ is a criterion that all known systems of criminal law recognise and seek to satisfy, G Fletcher, *The Grammar of Criminal Law, vol 1: Foundations* (OUP 2007) 222.

15 Abbott and Sarch (n 11) 328. Azzutti (n 1) discusses ‘hard cases’ of algorithmic market manipulation, and MA Lemley and B Casey, ‘Remedies for Robots’ (2019) 86 U Chi L Rev 1311 discuss ‘unforeseeable AI harms’ corresponding to ‘hard AI crime’.

16 A similar definition—without the requirement at point 4 below—is proposed by the Singapore Academy of Law, Law Reform Committee, *Report on Criminal Liability, Robotics and AI Systems* (2021) <www.sal.org.sg/sites/default/files/SAL-LawReform-Pdf/2021-02/2021%20Report%20on%20Criminal%20Liability%20Robotics%20%26%20AI%20Systems.pdf> accessed 11 March 2024.

17 Simester (n 9) ch 3.

18 GS Fletcher, ‘Deterring Algorithmic Manipulation’ (2021) 74 Vand L Rev 259.

19 Regulation (EU) No 596/2014 of the European Parliament and of the Council of 16 April 2014 on market abuse [2014] OJ L173/1.

20 Directive 2014/57/EU (Market Abuse Directive, hereinafter MAD) imposes criminal sanctions only against human traders who *intentionally* employ market manipulation techniques (Art 5(1) MAD).

21 Fletcher, ‘Deterring Algorithmic Manipulation’ (n 18) 288.

22 ibid 293.

23 For his involvement in the 2010 flash crash, Mr Sarao was sentenced in 2020 to a year of home incarceration: K Martin, ‘Flash Crash: The Trading Savant Who Crashed the US Stock Market’ *Financial Times* (7 May 2020) <www.ft.com/content/5ca93932-8de7-11ea-a8ec-961a33ba80aa> accessed 11 March 2024. His defence team argued that Sarao was on the autism spectrum, with Asperger’s syndrome, and that, to him, beating the markets was ‘like winning a video game’.

24 On *mens rea* as a necessary but not sufficient condition for establishing criminal responsibility, see J Edwards, ‘Theories of Criminal Law’, *The Stanford Encyclopedia of Philosophy* (Fall edn, 2021) <https://plato.stanford.edu/entries/criminallaw/> accessed 11 March 2024.

25 F Lagioia and G Sartor, ‘AI Systems under Criminal Law: A Legal Analysis and a Regulatory Perspective’ (2020) 33 Philosophy & Technology 433; Abbott and Sarch (n 11); H Ashton, ‘Definitions of Intent Suitable for Algorithms’ (2022) 31(3) Artificial Intelligence and Law 515. Without being able to go into detail here, Bratman’s Belief–Desire–Intention (BDI) Model of rational action, which emphasises the functional role of human intentions, is the conceptual framework used in these approaches to attribute an intentional mental state to AI agents whose architecture correspond to the BDI Model. M Bratman, *Intention, Plans, and Practical Reason* (Harvard UP 1987). Intentions are, essentially, plans of action to which the agent has committed, and this commitment is inferred from the fact that actors who intend to bring about an outcome guide their conduct in the direction of causing that outcome, irrespective of any internal disposition towards the outcome (whether they approve of it or understand its moral significance, etc). That matches reasonably well with the technical definition of intent in criminal law, where an intentional criminal conduct may as well be blameless because, for example, a child or a person claiming an insanity defence was the perpetrator.

26 Indicatively, Simester (n 9); Ashton (n 25); G Taylor, ‘Concepts of Intention in German Criminal Law’ (2004) 24 OJLS 99, 101.

27 Fletcher, ‘Deterring Algorithmic Manipulation’ (n 18) 297.

28 For an overview of the debate on the rationale of punishment, see H Zachary and A Duff, ‘Legal Punishment’, *The Stanford Encyclopedia of Philosophy* (Summer edn, 2022) <https://plato.stanford.edu/entries/legal-punishment/> accessed 11 March 2024; Fletcher, *The Grammar of Criminal Law* (n 14) 193–5.

29 M Moore, *Placing Blame: A Theory of Criminal Law* (OUP 1997).

30 HLA Hart, *Punishment and Responsibility: Essays in the Philosophy of Law* (2nd edn, OUP 1967).

31 J Gardner, ‘Introduction’ in Hart (n 30) xxiii–xxxi argues that punishment imposed to reduce *future* wrongdoing, and not to correct for *past* wrongdoing, does not even conceptually live up to its name, since punishment must by its nature be *for* an *actual *or *supposed*** wrong, even if the punishment need not be imposed on the actual or supposed wrongdoer.

32 Simester (n 9) 6.

33 ibid.

34 T Brooks, ‘Hegel on Crime and Punishment’ in T Brooks and S Stein (eds), *Hegel’s Political Philosophy: On the Normative Significance of Method and System* (OUP 2017) 210. Of course, we could treat AI agents ‘like dogs to be threatened’ as long as they lack ‘honour’ and ‘freedom’.

35 Simester (n 9) 11.

36 ibid 77.

37 JM Fischer and M Ravizza, *Responsibility and Control: A Theory of Moral Responsibility* (CUP 1998).

38 RA Duff, *Answering for Crime: Responsibility and Liability in the Criminal Law* (Bloomsbury, 2009; Simester (n 9).

39 S Russell, *Human Compatible: Artificial Intelligence and the Problem of Control* (Penguin, 2019) 16.

40 J Coleman, ‘Crimes and Transactions’ (1985) 88(3) CLR 921.

41 GS Becker, ‘Crime and Punishment: An Economic Approach’ (1968) 76(2) Journal of Political Economy 169.

42 A Harel, ‘Criminal Law as an Efficiency-Enhancing Device: The Contribution of Gary Becker’ in MD Dubber (ed), *Foundational Texts in Modern Criminal Law* (OUP 2014).

43 T Fisher, ‘Economic Analysis of Criminal Law’ in Markus D Dubber and Tatjana Hörnle (eds), *The Oxford Handbook of Criminal Law* (OUP 2014).

44 Without understating the resources of non-criminal law to achieve deterrence, eg punitive damages in civil law, taxation, etc, criminal law arguably remains the most efficient deterrence system, as it is also backed by its deontologically rooted aspects, eg signalling of moral condemnation. P Robinson, ‘The Criminal–Civil Distinction and the Utility of Desert’ (1996) 76 BU L Rev 201; R Williams, ‘Criminal Law in England and Wales: Just Another Form of Regulatory Tool?’ in M Dyson and B Vogel (eds), *The Limits of Criminal Law* (Intersentia 2021).

45 RA Posner, ‘An Economic Theory of the Criminal Law’ (1985) 85 Colum L Rev 1193.

46 For instance, if an AI trading agent were promptly ‘punished’ whenever its action causes market disruption, regardless of whether that is the agent’s intended outcome, the agent may be induced to abstain from many useful commercial practices, since circumstances may arise, however improbably, under which such practices may cause such disruption.

47 G Becker, *The Economic Approach to Human Behavior* (University of Chicago Press, 1976) 5.

48 D Cornish and RV Clarke, *The Reasoning Criminal: Rational Choice Perspectives on Offending* (Springer 1986).

49 The literature is vast and we cannot hope to do justice to it here. Indicatively, see TS Ulen and RH McAdams, ‘Behavioral Criminal Law and Economics’ (2008) University of Chicago Law and Economics Working Paper 440/2008 <https://papers.ssrn.com/sol3/papers.cfm?abstract_id=1299963> accessed 11 March 2024; PH Robinson and JM Darley, ‘Does Criminal Law Deter? A Behavioural Science Investigation’ (2004) 24 OJLS 173; N Garoupa, ‘Behavioral Economic Analysis of Crime: A Critical Review’ (2003) 15 EJLE 5. D Kahneman and A Tversky, ‘Prospect Theory: An Analysis of Decision Under Risk’ (1979) 47 Econometrica 263 proposed an alternative to the Subjective Expected Utility Theory called ‘Prospect Theory’, which was consistent with the behavioural evidence.

50 Harel (n 42).

51 Practically, this means that the optimal sanction should be increased by the reciprocal of the detection rate. For instance, if 1 in 10 criminals are caught, and the gains to be obtained through undetected crimes are €100, the optimal (monetary) sanction is (at least) €100 × 10 = €1000.

52 Posner (n 45) 1213 acknowledges the claim that selective enforcement of laws ‘creates ex-post inequality among offenders’, but he sees nothing wrong with this. In his view, the criminal justice system is like a lottery, which is voluntary and thus not unfair. One can keep out of the system by not committing crimes; if one commits a crime, one enters the lottery.

53 Lemley and Casey (n 15) 1384 argue that for ‘bad robots’ we should minimise the psychologically driven aspects of deterrence and replace them with more rational measures of cost.

54 J Edwards and A Simester, ‘Prevention with a Moral Voice’ in Simester, du Bois-Pedain and Neumann (eds), *Liberal Criminal Theory* (Hart Publishing 2014).

55 C Véliz, ‘Moral Zombies: Why Algorithms Are Not Moral Agents’ (2021) 36 AI & Society 487, 491.

56 DC Parkes and MP Wellman, ‘Economic Reasoning and Artificial Intelligence’ (2015) 349(6245) Science 267, 270 note that AI is in fact striving to construct a synthetic *homo economicus*, ‘perhaps most accurately termed *machina economicus*’. In the criminal law context, the applicability of criminal law and economics to the deterrence of rational AI agents has been recently touched upon by A Giannini, *Criminal Behavior and Accountability of Artificial Intelligence Systems* (Eleven Publishers 2023) ch 6.

57 S Russell and P Norvig, *Artificial Intelligence: A Modern Approach* (Pearson Education 2021) 22.

58 ibid 58.

59 ibid 23. Recall Mr Sarao’s defence team arguing that for him beating the markets was like ‘winning a video game’.

60 ibid 71.

61 On the compatibility of rationality and altruism, see AK Sen, *On Ethics and Economics* (Blackwell 1987).

62 The expected utility of an action *a* may be denoted, in a mathematical formulation, as EU(*a*) = SUM_*o*∈*O*, *s*∈S_ (P(*o*|*a,s*)U(*o*)), where P(*o*|*a,s*) is the probability that the outcome *o* will be produced if action *a* takes place in context *s*, and U(*o*) is the utility of outcome *o*. This means that we obtain the utility of *a* by summing up, for each possible outcome *o* and each possible context *s*, the results of multiplying the utility of *o* by the probability that *o* will be produced by performing action *a* in context *s*. For simplicity, we assume that for any action and state, it is either certain or impossible that the machine will achieve the outcome in that state: P(*o*|*a*,*s*) is either 1 or 0. For a discussion of different approaches to expected utility, see RA Briggs, ‘Normative Theories of Rational Choice: Expected Utility’, *The Stanford Encyclopedia of Philosophy* (Fall edn, 2019) <https://plato.stanford.edu/entries/rationality-normative-utility/> accessed 11 March 2024.

63 The selected action *a* is the one delivering the maximum utility, within the set *A* of all possible actions, which is usually denoted as argmax_*A*_ EU(*a*).

64 An economic argument against ‘draconian’ sanctions is that of ‘marginal deterrence’: if *all* sanctions are draconian, prospective criminals will not be deterred from committing more serious crimes, GJ Stigler, ‘The Optimum Enforcement of Laws’ (1970) 78 Journal of Political Economy 343.

65 P Lin, ‘Why Ethics Matters for Autonomous Cars’ in M Mauer and others, *Autonomous Driving* (Springer 2015). For a discussion, see G Contissa, F Lagioia and G Sartor, ‘The Ethical Knob’ (2017) 25(3) Artificial Intelligence and Law 365.

66 Examples are the ones contained in WD Ross, *The Right and the Good* (Clarendon Press 1930). On machine morality, see, among others, W Wallach and C Allen, *Moral Machines: Teaching Robots Right from Wrong* (OUP 2008); M Anderson and S Leigh Anderson (eds), *Machine Ethics* (CUP 2011); A Winfield and others, ‘Machine Ethics: The Design and Governance of Ethical AI and Autonomous Systems’ (2019) 107 Proceedings of the IEEE 507.

67 For a review, see H Prakken and G Sartor, ‘Law and Logic: A Review from an Argumentation Perspective’ (2015) 227 Artificial Intelligence 214.

68 T Bench-Capon and others, ‘Argument Schemes for Reasoning with Legal Cases Using Values’ (ICAIL 2013: Fourteenth International Conference on Artificial Intelligence and Law) 13–22; J Maranhao, E de Souza and G Sartor, ‘A Dynamic Model for Balancing Values’ (ICAIL 2021: Eighteenth International Conference for Artificial Intelligence and Law) 89–98.

69 As argued by G Calabresi and AD Malamed, ‘Property Rules, Liability Rules and Inalienability: One View of the Cathedral’ (1972) 85 Harv L Rev 1089.

70 The Holmesian ‘bad man’ concept has also been explored by Lemley and Casey (n 15) to conceptualise effective legal remedies for robots and is used by TD Grant and D Wischik, who draw an analogy between the legal philosophy of Oliver Wendell Holmes, Jr and the machine learning revolution in computer science. See TD Grant and D Wischik, *On the Path to AI: Law’s Prophecies and the Conceptual Foundations of the Machine Learning Age* (Palgrave Macmillan 2020).

71 OW Holmes, Jr, ‘The Path of the Law’ (1897) 10 Harv L Rev 457, 459.

72 B Casey, ‘Amoral Machines, or: How Roboticists Can Learn to Stop Worrying and Love the Law’ (2017) 111 Northwestern University Law Review 1347.

73 ibid.

74 This distinction would roughly correspond to criminal law’s distinction between *mala in se* (wrongs in themselves) and *mala prohibita* (wrongs established by statute).

## Notes

This research has received funding from the European Research Council (ERC) under the European Union’s Horizon 2020 research and innovation programme (Grant Agreement no. 833647). We would like to thank Fabrizio Esposito, Sabine Gless, Pekka Mäkelä, Nicolas Petit and Athina Sachoulidou for helpful comments at various stages.

